# An International Contrast of Rates of Placental Abruption: An Age-Period-Cohort Analysis

**DOI:** 10.1371/journal.pone.0125246

**Published:** 2015-05-27

**Authors:** Cande V. Ananth, Katherine M. Keyes, Ava Hamilton, Mika Gissler, Chunsen Wu, Shiliang Liu, Miguel Angel Luque-Fernandez, Rolv Skjærven, Michelle A. Williams, Minna Tikkanen, Sven Cnattingius

**Affiliations:** 1 Department of Obstetrics and Gynecology, College of Physicians and Surgeons, Columbia University, New York, New York, United States of America; 2 Department of Epidemiology, Mailman School of Public Health, Columbia University, New York, New York, United States of America; 3 THL National Institute for Health and Welfare, Helsinki, Finland and NHV Nordic School of Public Health, Gothenburg, Sweden; 4 Section for Epidemiology, Department of Public Health, Aarhus University, Aarhus, Denmark; 5 Health Surveillance and Epidemiology Division, Centre for Chronic Disease Prevention, Public Health Agency of Canada, Ottawa, Canada; 6 Department of Epidemiology, School of Public Health, Harvard University, Boston, Massachusetts, United States of America; 7 Department of Global Public Health and Primary Care, University of Bergen, Bergen, Norway; 8 Medical Birth Registry of Norway, Norwegian Institute of Public Health, Bergen, Norway; 9 Department of Obstetrics and Gynecology, University Central Hospital, Helsinki, Finland; 10 Clinical Epidemiology Unit, Department of Medicine Solna, Karolinska Institutet, Stockholm, Sweden; Central South University, CHINA

## Abstract

**Background:**

Although rare, placental abruption is implicated in disproportionately high rates of perinatal morbidity and mortality. Understanding geographic and temporal variations may provide insights into possible amenable factors of abruption. We examined abruption frequencies by maternal age, delivery year, and maternal birth cohorts over three decades across seven countries.

**Methods:**

Women that delivered in the US (n = 863,879; 1979–10), Canada (4 provinces, n = 5,407,463; 1982–11), Sweden (n = 3,266,742; 1978–10), Denmark (n = 1,773,895; 1978–08), Norway (n = 1,780,271, 1978–09), Finland (n = 1,411,867; 1987–10), and Spain (n = 6,151,508; 1999–12) were analyzed. Abruption diagnosis was based on ICD coding. Rates were modeled using Poisson regression within the framework of an age-period-cohort analysis, and multi-level models to examine the contribution of smoking in four countries.

**Results:**

Abruption rates varied across the seven countries (3–10 per 1000), Maternal age showed a consistent J-shaped pattern with increased rates at the extremes of the age distribution. In comparison to births in 2000, births after 2000 in European countries had lower abruption rates; in the US there was an increase in rate up to 2000 and a plateau thereafter. No birth cohort effects were evident. Changes in smoking prevalence partially explained the period effect in the US (P = 0.01) and Sweden (P<0.01).

**Conclusions:**

There is a strong maternal age effect on abruption. While the abruption rate has plateaued since 2000 in the US, all other countries show declining rates. These findings suggest considerable variation in abruption frequencies across countries; differences in the distribution of risk factors, especially smoking, may help guide policy to reduce abruption rates.

## Introduction

Roughly a third of all pregnancies in high-income countries are diagnosed with some medical or obstetrical complication, contributing to the substantial burden of maternal and perinatal morbidity and mortality. Placental abruption, one of the severest of all obstetrical complications, is a condition when the placenta detaches prematurely [1]. The complication is associated with disproportionately high rates of perinatal mortality and morbidity and neurodevelopmental deficits in children later in life [2–5]. Women diagnosed with abruption suffer 3 to 4-fold increased risk of premature cardiovascular mortality and, morbidity [6,7].

The prevalence of abruption in European countries is 3–6 per 1000 pregnancies [8–10], whereas the corresponding data in North America is two-fold higher (7–12 per 1000 pregnancies). Furthermore, recent European studies have documented a temporal decline in abruption rates [9,11], but data from the US and, Canada have shown increased rates [12–14]. The change in the prevalence rate of abruption and the contrasting geographical heterogeneity in both rates and trends suggest that population-based risk factors for abruption may contribute to differences in rates. An international contrast of abruption rates using an age-period-cohort (APC) analysis may provide insights toward an understanding of how and why abruption trends change over time.

There is substantial empirical evidence that supports a potential contribution of age, period, as well as cohort effects to abruption rates. Maternal age shows a J-shaped distribution of risk for abruption, with young (<20 years) and advanced maternal age (≥35 years) at increased risk [15]. By period and cohort, temporal trends in risk factors for abruption such as cigarette smoking [16,17] may be contributing to the observed temporal variation. Smoking during pregnancy is a strong and consistently observed risk factor for abruption [4,12,17–21]. With the exception of Finland where the prevalence of smoking during pregnancy has remained stable at 15%, smoking has generally decreased in the US, Canada, Sweden, Denmark and Spain in the last two decades [22–25].

In a large, population-based study of births in the US, Canada, Sweden, Norway, Denmark, Finland and Spain, we examine abruption rates across three decades with regard to maternal age, time of occurrence of abruption and maternal year of birth (cohort). We hypothesize a decline in birth cohort effects on abruption, largely driven by the declining prevalence of smoking.

## Methods

### Study design and data sources

We implemented a population-based, cohort analysis of births over three decades in the United States, Canada, Sweden, Norway, Denmark, Finland and Spain. The study was restricted to women delivering a singleton live birth or stillbirth (with the exception of Denmark that included only live births).

### Ethics statement

Since data for all countries are de-identified, they do not qualify as human subjects research; therefore, no ethics approval from an Institutional Review Board was sought for this study.

### United States births, 1979–2010

Data for US births were derived from the 1979–2010 National Hospital Discharge Survey (NHDS) datasets, assembled by the US Centers for Disease Control and Prevention. These data are based on hospitalizations from short-stay (<30 days) hospitals, and non-federal general and specialty hospitals. The data included in the survey are representative of sample of hospital discharges in the 50 states and the District of Columbia.

Between 1979 and 1987, the survey used a two-stage stratified sampling design; since 1988 the survey used a modified three-stage design [26,27]. The number of hospitals surveyed each year ranged between 400 and 558 and included approximately 181,000 to over 300,000 hospital discharges each year. Beginning in 2008, the number of hospitals chosen for the survey was reduced by half (roughly 200 to 300 hospitals). When the sampling weights are applied, these data represent all in-patient hospitalizations in the US. All diagnoses and procedures were coded based on the International Classification of Disease (ICD), 9^th^ revision, Clinical Modification.

### Canada births, 1981–2010

Data on Canadian births were abstracted from the Canadian Institute for Health Information’s hospital Discharge Abstract Database (DAD). The DAD contains data on every hospitalization, including demographic and residence information, length of stay, diagnoses and procedures performed during the hospitalization. Up to 2000–01 all diagnoses were coded according to ICD-9-CM, and procedures were coded according to the Canadian Classification of Diagnostic, Therapeutic, and Surgical Procedures. Since 2001, the ICD-10 and the Canadian Classification of Health Interventions for procedures were adopted by the participating hospitals. The DAD includes data on deliveries of all live births and stillbirths. Only hospitalizations occurring in the provinces of Prince Edward Island, Ontario, Saskatchewan, and British Columbia were complete in the DAD over the entire period of study (accounting for over 50% of the whole Canadian population), and were analyzed in this study [28,29].

### Sweden births, 1978–2010

Data were derived from the nation-wide Swedish Medical Birth Register (SMBR), maintained by the National Board of Health and Welfare. Data in the SMBR is collected through standardized prenatal, obstetric and neonatal records, which are forwarded to the registry after delivery. The SMBR includes information on more than 98% of births in Sweden [30]. All diagnoses and procedures were coded based on the Swedish versions of the ICD-8 (1978–1986), ICD-9 (1987–1996) and ICD-10 (1997 and later) revisions.

### Norway births, 1978–2009

Based on compulsory notification, the Medical Birth Registry of Norway (MBRN) has since 1967 recorded medical data on all births in Norway with a gestational age of at least 16 weeks, including data on maternal disease and complications during pregnancy. The attending midwife and doctor using a standardized notification form, either as free text or by predefined variables or check boxes, register data. Free text is coded at the MBRN using ICD-8 revision for years 1967–98 and ICD-10 since 1999. MBRN contains all births in Norway for all years since 1967.

### Denmark births, 1978–2008

Data for Danish births were derived from the Danish Birth Register and the Danish Hospital Register. We identified all live-born infants between 1978 and 2008 from the Danish Medical Birth Register, which includes all births in Denmark since 1973 [31]. All live-born infants and new residents in Denmark are assigned a unique civil registration number, which were used to link information from several national registries at the individual level. Data on abruption was extracted from the Danish National Hospital Register that holds nationwide data on all admissions to any Danish hospital since 1977 and on all outpatient visits since 1995. The discharge diagnoses are based on the Danish version of the ICD-8 (1977–1993), and ICD-10 (1994–2008) [32].

### Finland births, 1987–2010

The linked Finnish Hospital Discharge Register (FHDR) and the Medical Birth Register (FMBR) were used to identify women with and without an abruption diagnosis based on the ICD-9 (1987–1995) or ICD-10 (1996–2010) codes in the FHDR and a check box in the FMBR (since October 1990). The FHDR and FMBR were linked using women’s unique encrypted identification number. The FMBR (since 1987) collects baseline data on health care and interventions women receive during pregnancy and delivery and newborn outcome during the first 7 days (<0.1% of newborns are missing in the FMBR). For all variables used in this study, the data have been validated and correspond well with information available in hospital records. The HDR collects information on inpatient episodes (since 1969), outpatient surgical procedures (since 1994), and outpatient visits in public hospitals (since 1998) [33,34].

### Spain births, 1999–2012

Data were derived from the National Institute of Statistics (NIS) in Spain. We used the vital-statistics database, for which the official data source is the standardized birth-registration form. This database contains information on all the births in Spain (http://www.ine.es). Data regarding diagnoses were derived from the Department of Health. We used the Spanish hospitals discharge database with codes based on the Spanish versions of the ICD-9-CM (1999–2012). These data files contain data on all public hospitals in Spain (www.msssi.gob.es/estadEstudios/estadisticas/cmbd/informes/notasMetodologicas.htm; accessed 1 April 2014).

### Classification and diagnosis of placental abruption

There are no standard or universally acceptable diagnostic criteria for placental abruption. The clinical criteria for an abruption diagnosis includes painful bleeding accompanied with tetanic uterine contractions or fetal heart rate decelerations; or evidence of blood clots embedded on the placental surface; or evidence of retroplacental bleeding behind the placental margin [35]. The ICD codes that were used to extract data on abruption included ICD-8 6321 and 6514; ICD-9 6412A and 6412B; and ICD-10 O450, O458 and O459.

### Age-period-cohort models

Age, period, and cohort (APC) effects were modeled using the classical approach for tabular data developed by Clayton and Shifflers [36,37]. Age, period, and cohort are linearly dependent variables (Cohort = Period-Age), thus modeling strategies require typically focus on capturing non-linear variation in tabular rates over time. Given the strong age effects associated with abruption, we first estimate abruption rates as a function of age. Then, we include a parameter for the overall linear change in abruption rates over time (termed “drift”) that is a sum of both period and cohort effects but not uniquely attributable to either. Finally, unique coefficients for period and cohort effects (termed “curvatures”) are obtained and interpreted as the second-order derivatives of the drift parameter.

The best fitting model of the data is selected by assessing the change in model fit (assessed by the likelihood-based deviance statistics and penalizing additional degrees of freedom) over successive models, beginning with an age parameter only, then age + drift, then the curvature of period given age and drift, etc. The modeling strategy was determined *a priori*, and was implemented with the function “*apc*.*fit”* in the “*Epi*” package [38] in the R software (version R.3.0.3; R Foundation for Statistical Computing, Vienna, Austria). Data from each country was analyzed separately.

### Sensitivity analysis

#### Effects of changes in population rates of cigarette smoking

While the above strategy allows for rigorous estimation of APC effects, these models do not allow for the incorporation of covariates, such as smoking, that may explain the observed APC effects in abruption. Therefore we fit multilevel models that allowed for covariates to predict changes in the period and cohort effect estimates of abruption over time [39]. Period and cohort were considered cross-classified random effects, and the aggregate smoking prevalence by country was tested to determine whether it explained between-group variance in period and cohort parameters.

The annual prevalence of smoking during pregnancy (yes/no) was available from five countries: US, Canada, Sweden, Norway and Finland. Data on smoking among US women (aged ≥15 years) giving birth each year was abstracted from the vital statistics data since 1989 (the year when smoking data were ascertained on birth certificates). Smoking data in Canada since 1994 were obtained from the National Population Health Survey and the Canadian Community Health Survey conducted by Statistics Canada [40]. Data on smoking in Sweden, Norway and Finland were ascertained at the first prenatal visit.

In the US, smoking decreased between 1989 (19.5%) and 2010 (9.2%); in Canada, from 35.5% in 1994 to 21.3% in 2009; in Sweden, from 30.9% in 1982 to 6.5% in 2010; and in Norway from 18.9% in 1999 to 7.5% in 2009. In Finland, the smoking prevalence remained constant between 1987 (15.0%) and 2010 (15.2%).

#### Analyses stratified based on maternal race

Given the well-known race disparity in abruption in the US [41], we examined if APC effects on abruption rates differed based on maternal race in the US. This sub-analysis was restricted to white (n = 468,257) and black (n = 119,466) women.

## Results

### Trends in prevalence rates of placental abruption

**Fig 1:**
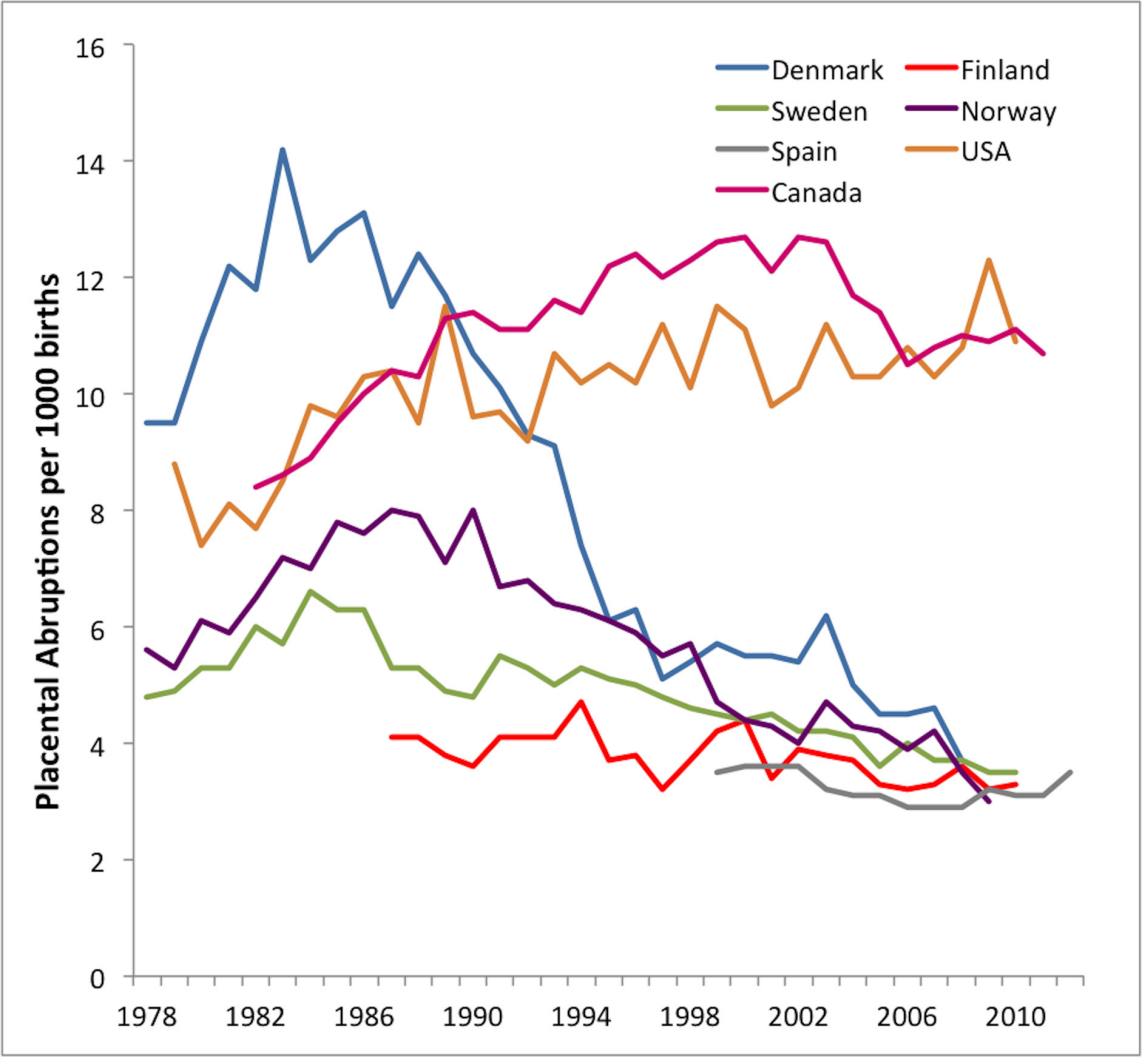
Temporal changes in the prevalence rates of placental abruption between 1978 and 2012 in the US, Canada, Sweden, Norway, Denmark, Finland and Spain.

The rate of abruption, across all years, was highest in the US (ranging from 7.4 in 1980 to 11.9 per 1000 in 2007) and lowest in Finland (ranging from 4.8 in 1994 to 3.3 per 1000 in 2009). The rates of abruption in all European countries showed a temporal decline, and the most dramatic decline was seen in Denmark (Fig 1). In North America, abruption rates increased in Canada while the rate in the US fluctuated over time, but overall there was an increasing trend over time also in the US.

**Fig 2:**
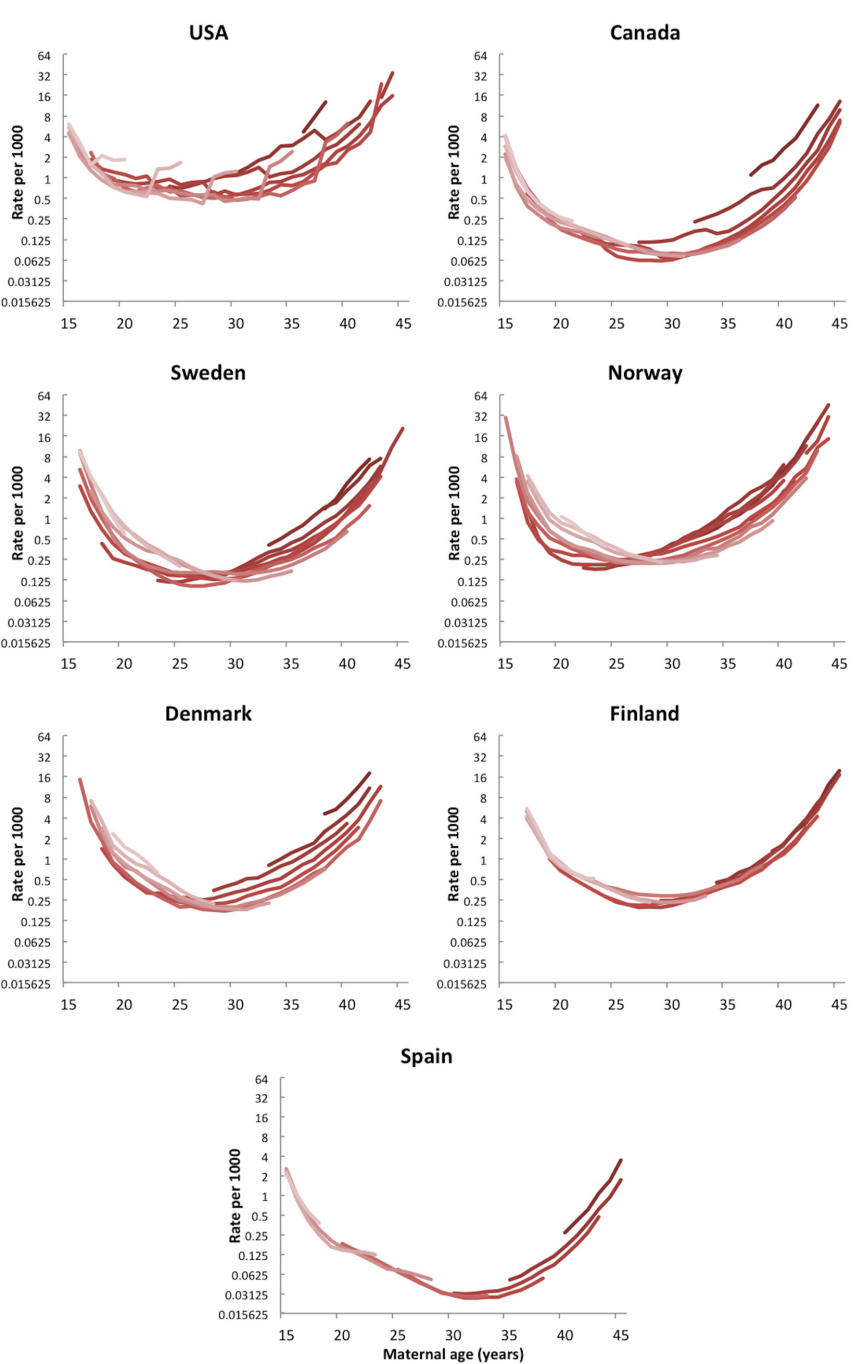
Rates of placental abruption in relation to maternal age within strata of maternal birth cohorts in the US, Canada, Sweden, Norway, Denmark, Finland and Spain.

Fig 2 shows the prevalence of abruption by maternal age, with each line reflecting a birth cohort. For ease of visual interpretation, we present every fifth cohort, and increasing darkness of the line represents increasingly younger cohorts. In all countries, there is a J-shaped distribution, with the highest rates among the youngest and oldest mothers. This shape is less pronounced in the US, wherein there is more overall variability in the rates. Further, these graphs reveal the appearance of cohort effects in Canada, Sweden, Norway, Denmark, and Spain, as the rates across age increase with increasingly younger cohorts. There is little evidence of cohort effects in Finland and in the US.

### Trends in placental abruption by risk factors

In Table 1 we present abruption rates in two years, 1981 (1987 in Finland) and 2008, by maternal age. Among women <20 year old, across all countries except Canada and Sweden, the rate decreased between the two periods; the largest relative decline in rate was in Denmark, and the smallest in Spain. Between 1981 and 2008, abruption rates among women <20 years old increased in both Canada and Sweden. There was a concomitant increase in abruption rates among women aged ≥35 years in the US, Canada, and Finland, and a decline in Sweden, Norway, Denmark and Spain.

**Table 1:** Changes in the prevalence of placental abruption based on maternal age in each country.

Maternal age	Period	Prevalence rate of placental abruption (per 1000 births)						
		USA n = 863,879	Canada n = 5,407,463	Sweden n = 3,266,742	Norway n = 1,780,271	Denmark n = 1,773,895	Finland = 1,411,867	Spain n = 6,151,508
**<20 years**	1981	9.4	9.9	3.4	6.2	15.8	5.7**†**	3.9**‡**
	2008	5.2	10.2	4.5	3.4	5.1	2.8	3.4
	Change (%)	-44.8	3.3	31.1	-45.9	-67.8	-50.5	-44.8
**≥35 years**	1981	11.2	10.5	11.1	7.4	11.8	4.6**†**	3.9**‡**
	2008	14.3	12.5	4.5	5.7	4.2	5.6	3.1
	Change (%)	27.7	19.1	-59.6	-23.5	-64.3	23.2	27.7

† Estimates are for 1987

**‡** Estimates for 1999. Change is estimated as the difference in abruption rates between the two periods relative to the rate in the earlier year

### Age, period, and cohort effects in placental abruption

APC model fit statistics are shown in Table 2. For all countries except Finland, a model with age, drift, as well as period and cohort curvatures provided the best fit to the data. In Finland, there was no significant change in model fit for any model, indicating that rates are best described by a single age parameter.

**Table 2:** Comparison of age-period-cohort sub-models for placental abruption prevalence rates.

Model parameter	Effect	Change in deviance (change in degrees-of-freedom)						
		USA	Canada	Sweden	Norway	Denmark	Finland	Spain
**Age-drift**	Δ†|A	25.6 (1)*	27.9 (1)*	394.2 (1)*	410.7 (1)	1622.6 (1)*	32.1 (1)*	33.5 (1)*
**Age-cohort**	P‡|A,C	4.1 (4)	64.3 (5)*	48.4 (4)*	62.4 (4)*	84.6 (4)*	1.4 (4)	-225.5 (5)*
**Age-period-cohort**	C‡|A,P	20.3 (5)*	375.1 (5)*	60.6 (5)*	2.0 (5)	342.0 (4)*	4.6 (4)	-568.8 (5)*
**Age-period**	P‡|A	-7.6 (-5)	-34.7 (-6)*	-9.3 (-5)	197.6 (-5)*	-6.8 (-4)	-1.9 (-4)	869.5 (-6)*
**Age-drift**	C‡|A	-16.8 (-4)*	-404.7 (-4)*	-99.6 (-4)*	-262.0 (-4)*	-419.8 (-4)*	-4. 1 (-4)	-75.1 (-4)*

* P <0.001. A, Age effect; P, Period effect; C, Cohort effect

†, Drift estimate

‡, Curvature estimate

**Fig 3:**
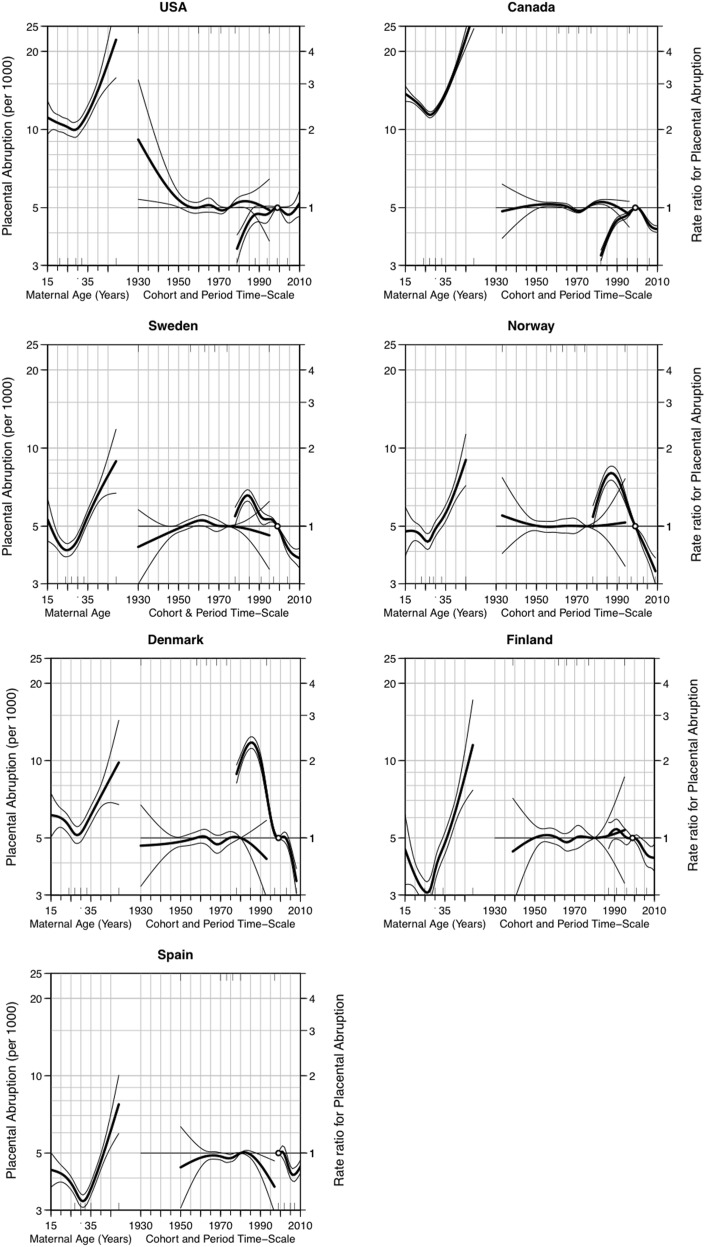
Age-period-cohort effects of placental abruption in the United States, Canada, Sweden, Norway, Denmark, Finland, and Spain. Abruption rates (per 1000) based on maternal age are shown on the left axis, and the rate ratio with 95% confidence interval for placental abruption in relation to maternal birth cohort (year 1975 as the reference) and period (year 2000 as the reference) are shown on the right axis.

Fig 3 shows the estimates of age, period, and cohort effects on abruption. The age-specific rates are plotted on the left axis, and period and cohort estimates are plotted on the rate ratio scale (right axis), with 2000 as the reference group for period and 1975 as the reference for birth cohort.

Similar to descriptive analysis, maternal age demonstrates a J-shaped relationship with abruption. Of note is that while the overall abruption rate in Denmark (averaged over 1978 and 2008) was 8 per 1000, the rates displayed in Fig 3 indicate that the abruption rate was, on average, 5 per 1000. This is likely consequence of strong confounding due to birth cohorts. The separation of a birth cohort effect from age and period shows an absence of cohort effect on abruption rates. In all seven countries, there was a slight temporal increase in the rate of abruption in the 1980s, and either a flattening of the rate (in the US) or a temporal decline, especially among the European countries.

### Sensitivity analysis

#### Changes in smoking as a potential explanatory factor

Using multi-level random effects models, we estimated whether changes in the prevalence of smoking over time explain variance in time-trends in abruption. We found that smoking prevalence significantly explained, at least in part, the between-period variation in abruption in the US (β = 0.05, 95% confidence interval 0.03, 0.10 and in Sweden (β = 0.16, 95% confidence interval 0.09, 0.31), and coefficients indicated that smoking rates are positively associated with period effects, suggesting higher rates of abruption in time periods in which there is a higher prevalence of smoking (S1 Fig). Smoking was not associated with between-group variance in Canada, and multi-level models for Norway and Finland did not converge.

APC models were also estimated by race in the US; results were consistent with the overall analysis for US (S2 Fig). There is a J-shaped relation between age and abruption, and no evidence of a decline in abruption rates by period similar to what was demonstrated in European countries among either White or Black mothers in the US.

## Discussion

The central goal of this international comparison is to evaluate the interactions of maternal age, period and birth cohorts on temporal changes in the rates of abruption. Unlike the US, all other countries show a clear decrease in abruption rates since 2000 or earlier, and in the US the rates have plateaued since 2000. These findings suggest that population rates of abruption are country specific and may be related to differences in the distribution of risk factors across countries. There is a strong and consistent maternal age effect on abruption. We document that changes in smoking prevalence are associated with changes in abruption rates in the US and Sweden.

### Limitations of the data

There are a few limitations to this international comparison of abruption rates. Importantly, selective fertility – the necessity of couples to replace a pregnancy loss [42,43] – may have influenced these APC effects on abruption to some extent. Women with abruption that result in a perinatal death are likely to replace the loss more quickly than women without any losses, leading to short interval between pregnancies (resulting in folate depletion). This, in turn, may increase the risk of abruption. Second, we were unable to account for repeat pregnancies within women and the possibility of increased recurrence of abruption across successive pregnancies [10,44,45]. A third limitation is the effect of risk factors that may account for the abruption trends, notably maternal behavioral and sociodemographic factors. However, adjustments for these factors is unlikely to modify the associations because the prevalence of sociodemographic factors are unlikely to exhibit similar trends over time as is seen in abruption rates. Fourth, a secular increase in the use of ultrasound to diagnose abruptions may have influenced the APC trends.

Misclassification of abruption may have affected our findings to some extent and such misclassification proportions probably vary across countries. In the US, for instance, the validity of abruption diagnosis in the NHDS data contrasted against medical chart abstraction was fairly high (sensitivity and positive predictive values of 89% each) [46]. In Finland, the validity in the FMBR and FHDR is high (97.8% agreement for diagnoses related to pregnancy, childbirth and puerperium). The possibility of coding differences across ICD classification between ICD-8 and ICD-10 may have had some impact on our findings. The APC modeling of abruption rates can also be subjected to residual confounding due to both measured and unmeasured factors. While some potential explanatory variables are common across all countries (e.g., maternal smoking), many are not (e.g., African-American race in the US where the abruption risk is 1.8-fold higher than non-Hispanic White women). Therefore, our approach was to focus on the international comparison that is population-based; we only adjusted for changes in the prevalence of smoking. Moreover, our adjustment for maternal smoking, at least in the US, was based on smoking prevalence rates by year. Since patient-level smoking data were unavailable (in the US), we cannot estimate the rate of abruption among women who smoke during pregnancy versus those who do not. However, our goal in the sensitivity analysis was to estimate the extent to which the overall population trends in smoking are associated with population trends in abruption, rather than to estimate individual-level risks of abruption associated with smoking status.

Appropriate APC modeling is an active area of methodological research. Every model is subject to the limitations of the assumptions of the technique. In our technique, we assume that age, period, and cohort have a linear effect on the outcome that is unobservable, and thus estimate deviations from linearity. We also triangulate results across two modeling strategies, including estimating drift and curvatures through Poisson regression as well as cross-classified random effects models. We do not account for non-linear associations among the variables, such as age by cohort or age by period interactions. However, the graphical data did not suggest that such interactions are likely to play a critical role in these estimates.

### Strengths of the study

With the exception of births in the US, this study includes virtually all singleton live births and stillbirths in Sweden, Norway, Denmark (live births only), Finland, and Spain and births in Canada are restricted to four provinces (Prince Edward Island, Ontario, Saskatchewan, and British Columbia). Thus, the study offers the potential for findings to be generalizable to countries with a similar distribution of risk factors. We employed a rigorous modeling strategy to provide a comprehensive overview of age, period, and cohort effects for each country, providing more nuance than traditional analyses of prevalence trends over time.

### Biologic interpretations

Maternal age is strongly associated with abruption, with young and especially older women at substantially increased risk. Both social and biologic factors are likely to have played a role in shaping abruption risk with respect to its association with maternal age. The increased abruption risk among young women is likely influenced by socioeconomic factors. Young women are less educated and of lower socioeconomic strata contributing to poor health. In turn, these factors also adversely affect pregnancy, thereby increasing their abruption risk. On the other hand, the strong association between increased maternal age and abruption is deeply rooted in the biologic underpinnings. Placental underperfusion and uteroplacental ischemia – strong contributors to abruption [47,48] – are commonly found in women of advanced age [49,50]. These women also carry less chance of successful reproduction, possibly due to aging uterine effects due to repeated pregnancies. In sum, the strong effect of increased age on abruption risk, independent of cohort and period effects, may reflect a pure biologic phenomenon is the consequence of strong selection of high-risk mothers with advanced age or both.

With the exception of Canada and the US, abruption rates have been declining in all the other countries in the 2000 (or earlier). In the US, the abruption rate initially increased up to 2000 and plateaued thereafter. One possible factor to explore in future studies in the fortification of foods with folate, which occurred in the US and Canada in 1998 and may have led to the decline (or plateauing) of abruption rates [51–54]. Epidemiologic studies have reported that women that took folate supplementation before or during the first trimester had a reduced risk of abruption [54]. Such universal folate fortification policy has not been implemented in European countries. However the increased general awareness of the benefits of multivitamins and folate in the prevention of neural tube defects and other pregnancy-related complications may have led women to consume folate prepregnancy or during pregnancy.

Smoking during pregnancy is a strong risk factor for abruption (RRs 1.7 to 3.0), and may have impacted abruption trends. Through a multilevel model, we tested if the recent decline in abruption rates were the result of the declining smoking prevalence rates. Indeed, at least in the US and Sweden, changing smoking rates were associated with between-period variation in abruption rates, offering at least a partial explanation for the period effects observed in these countries. While smoking does not fully explain the temporal decline in abruption rates, the temporal decline in smoking rates may have helped in muting the increase in abruption rates.

What other factors might have contributed to the observed temporal decline in abruption rates? Among the many risk factors that are associated with abruption [19,50,55], changes in diagnostic criteria for abruption may have also played a role. There is no standardized definition for abruption [35]. It is therefore unlikely that differences in diagnostic criteria for abruption may have led to differences in the prevalence rates of abruption across countries. We did not detect a birth cohort effect associated with abruption. This suggests that the population factors contributing to changes in abruption rates are those that have affected women of childbearing age, rather than factors that have varied across age groups.

## Conclusions

The strong maternal age effect on placental abruption is consistent across all seven countries in this international comparison; these data do not show any evidence of a birth cohort effect. Since 2000 (or earlier), abruption rates in Canada, Sweden, Norway, Denmark, Finland and Spain show a sharp decline but the rates in the US since the 2000 have plateaued. These observations suggest that factors that contribute to abruption may differ across countries. The independent effects of maternal age and, to a lesser extent, period of birth, coupled with differences in these determinants across countries point to the complicated dynamics that shape abruption risk over time.

## Supporting Information

S1 Fig Parameter estimates for smoking as a predictor of period effects for placental abruption in three countries: Sweden, United States, and Canada. (DOCX)

S2 Fig Age-period-cohort effects of placental abruption among White and Black women in the United States. (DOCX)
