# Supplementary material for: An International Contrast of Rates of Placental Abruption: An Age-Period-Cohort Analysis
Source: PLoS One. 2015 May 27;10(5):e0125246. doi: 10.1371/journal.pone.0125246 (PMC4446321; doi:10.1371/journal.pone.0125246)
Supplement: S1 Fig — (DOCX) [file pone.0125246.s001.docx]

**Supplemental figure 1**

**Parameter estimates for smoking as a predictor of period effects in three countries: Sweden, United States, and Canada**

**
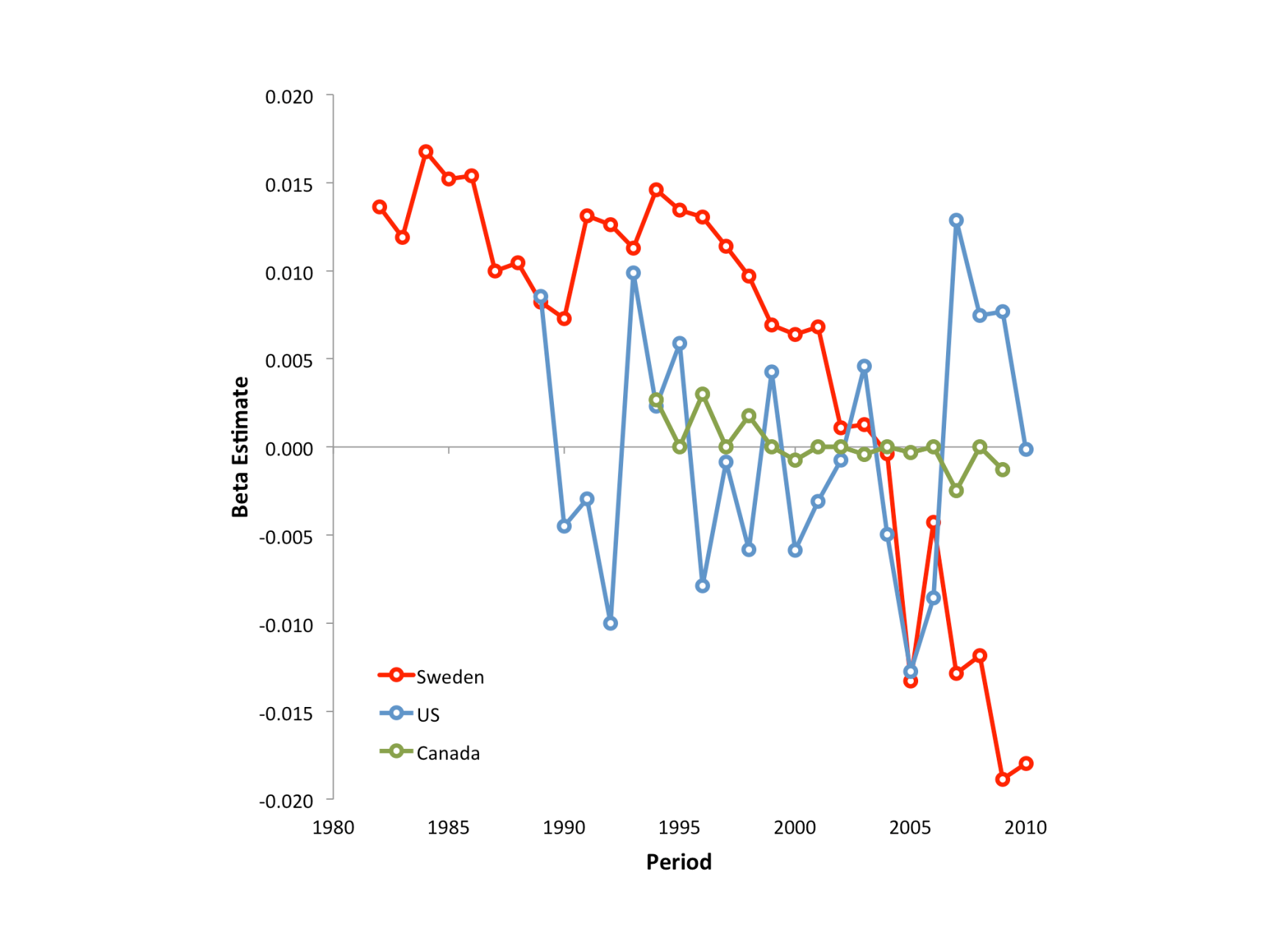
**
